# Finite Temperature String with Order Parameter as Collective Variables for Molecular Crystal: A Case of Polymorphic Transformation of TNT under External Electric Field

**DOI:** 10.3390/molecules29112549

**Published:** 2024-05-29

**Authors:** Shi-Jie Niu, Fu-De Ren

**Affiliations:** 1 School of Chemistry and Chemical Engineering, North University of China, Taiyuan 030051, China; niushijie1995_asdf@126.com; 2 School of Management, Wuhan Polytechnic University, Wuhan 430040, China

**Keywords:** molecular crystal, polymorphic transformation of TNT, finite temperature string, order parameters, external electric field

## Abstract

An external electric field is an effective tool to induce the polymorphic transformation of molecular crystals, which is important practically in the chemical, material, and energy storage industries. However, the understanding of this mechanism is poor at the molecular level. In this work, two types of order parameters (OPs) were constructed for the molecular crystal based on the intermolecular distance, bond orientation, and molecular orientation. Using the K-means clustering algorithm for the sampling of OPs based on the Euclidean distance and density weight, the polymorphic transformation of TNT was investigated using a finite temperature string (FTS) under external electric fields. The potential of mean force (PMF) was obtained, and the essence of the polymorphic transformation between *o*-TNT and *m*-TNT was revealed, which verified the effectiveness of the FTS method based on K-means clustering to OPs. The differences in PMFs between the *o*-TNT and transition state were decreased under external electric fields in comparison with those in no field. The fields parallel to the *c*-axis obviously affected the difference in PMF, and the relationship between the changes in PMFs and field strengths was found. Although the external electric field did not promote the convergence, the time of the polymorphic transformation was reduced under the external electric field in comparison to its absence. Moreover, under the external electric field, the polymorphic transformation from *o*-TNT to *m*-TNT occurred while that from *m*-TNT to *o*-TNT was prevented, which was explained by the dipole moment of molecule, relative permittivity, chemical potential difference, nucleation work and nucleation rate. This confirmed that the polymorphic transformation orientation of the molecular crystal could be controlled by the external electric field. This work provides an effective way to explore the polymorphic transformation of the molecular crystals at a molecular level, and it is useful to control the production process and improve the performance of energetic materials by using the external electric fields.

## 1. Introduction

Recently, the external electric field has been as an effective tool to induce the variation in phase transformation [1,2], with the formation of the potential new crystal phase and morphology so as to realize the control and optimization of the production process and improve product quality [3,4,5,6,7]. 

The polymorphism of molecular crystals is a phenomenon in which the system with a definite molecular composition can transform into the different crystal forms with the different physical or chemical properties, and it is important practically in the chemical, material, and energy storage industries, etc. [8,9,10,11,12,13,14]. Exploring the polymorphic transformation mechanism at the molecular level is essential for effectively introducing an external electric field to control the transformation products and improve crystal qualities [1,5,7]. However, despite extensive experimental [12,13,14,15,16,17,18,19,20] and theoretical [21,22,23] investigations on the polymorphic transformation of the molecular crystals, at present it is still unclear how the molecules transform into the new crystal forms under the external electric field [1,3,4,5,6]. Due to the much lower time scale than that of real occurrence [24,25], it is difficult to converge free energy in the polymorphic transformation, as it is a rare event [26,27] in theoretical simulation [28]. Furthermore, due to the sophisticated molecular structure, it is difficult to find a set of suitable collective variables which are used to distinguish two polymorphs and track the transformation path [26]. 

Finite temperature string (FTS), as an effective rare event method, can be used to simulate the phase transformation process [29,30,31,32]; and, by the reparameterization of the images along the path of the phase transformation [30], the minimum-free energy path (MFEP) could be obtained with the string method in collective variables (SMCV) [33]. Therefore, various FTS methods have been recently developed to explore the MFEPs along the phase transformation [34,35,36,37,38,39]. In order to find out a set of suitable collective variables used in the simulation of the phase transition for the molecular crystal, Santiso [26] and Bellucci et al. [40] constructed the order parameters (OPs) only by using the data from the molecular dynamics (MD) simulation. Thus, an MFEP connecting the different crystal states could be obtained by combining OPs with SMCV to monitor the nucleation of the molecular crystals at the molecular level [41,42]. 

K-means clustering is an algorithm by which a data set containing *n*-dimensional vectors are clustered and divided into *k* sub-clusters by the iterations [43,44]. As a new enhanced sampling algorithm, it has been used in machine learning [45,46]. Recently, we used the K-means clustering algorithm based on the Euclidean distance and sample weight to improve the convergence of FTS with OP in the MFEP connecting between the molecular crystals *β*-CL-20 and *ε*-CL-20 [47]. For the ionic crystal, we carried out a theoretical investigation on the external electric-field-induced crystallization of TKX-50 using FTS, with the K-means clustering to the sampling of OPs [48]. However, to our knowledge, no theoretical investigation into the polymorphic transformation of the molecular crystal using the FTS method with the K-means clustering to the sampling of OPs under the external electric field has been made. 

It is very important to reveal the polymorphic transformation mechanism of explosives for the molecular design and synthesis of the high energetic and insensitive materials [49,50,51,52,53]. TNT (2,4,6-Trinitrotoluene) is the most widely used melting and casting explosive (see Figure S1). It has two kinds of the polymorphism; these are orthorhombic and monoclinic forms, referred to as *o*-TNT and *m*-TNT [54]. 

Following our recent investigation on the polymorphic transformation of the ionic crystal using the FTS method under the external electric field [48], in this work, the polymorphic transformation involving the molecular crystal TNT was present. The local OPs of *o*-TNT and *m*-TNT were constructed using the structural variables involving the bond distances, bond orientations and relative orientations. A K-means clustering algorithm based on the dimensionally weighted Euclidean distance and sample weight was used to construct the smooth initial FTS. A MFEP connecting *o*-TNT and *m*-TNT was sketched by using the SMCV method, and then the potential of mean force (PMF) was calculated under the external electric field. At the molecular level, the essence of the polymorphic transformation between *o*-TNT and *m*-TNT was revealed under the external electric field. This study extends the application scope of FTS in the investigation into the polymorphic transformation of explosive molecular crystals using the K-means clustering technique with OPs as the collective variables, and it is useful for further optimizing the technological process to obtain the desired polymorph of the explosive molecular crystals under the external electric field in experiment. 

## 2. Theory 

### 2.1. Order Parameter

For the molecular crystals, the order parameter (OP) is a set of mathematical functions containing the intermolecular distance, bond orientation, relative orientation, and internal configurations, etc., and the change in OPs means the change in the translation, vibration and rotation of the functional groups as well as the rearrangement of molecules [26]. The OPs of the bond distance combining with the bond orientation or relative orientation, signed as $\varphi_{\alpha ,i}^{\mathrm{db}}$ and $\varphi_{\alpha ,i}^{\mathrm{dr}}$, can be used to describe the polymorphic transformation of the molecular crystal involving the molecules with high planar symmetry [26]. They can be defined as:

(1) $$\varphi_{\alpha ,i}^{\mathrm{db}}=\frac{1}{\sqrt{2\pi }\sigma_{\alpha }}\frac{1}{2\pi I_{0}\left({\eta_{\hat{r}}}^{\alpha }\right)}\sum_{j\neq i}\exp\left[-\frac{{\left({\hat{r}}_{ij}-{\hat{r}}_{\alpha }\right)}^{2}}{2\sigma_{\alpha }^{2}}\right]\exp\left[{\eta_{\hat{r}}}^{\alpha }\cos2\left(\phi_{\hat{r}}-\phi_{\hat{r}}^{\alpha }\right)\right]$$

(2) $$\varphi_{\alpha ,i}^{\mathrm{dr}}=\frac{1}{\sqrt{2\pi }\sigma_{\alpha }}\frac{1}{2\pi I_{0}\left(\eta_{q}^{\alpha }\right)}\sum_{j\neq i}\exp\left[-\frac{{\left({\hat{r}}_{ij}-{\hat{r}}_{\alpha }\right)}^{2}}{2\sigma_{\alpha }^{2}}\right]\exp\left[\eta_{q}^{\alpha }\cos2\left(\phi_{q}-\phi_{q}^{\alpha }\right)\right]$$

where *r_ij_* denotes the distance between the centers-of-mass of two molecules *i* and *j*, $\phi_{\hat{r}}$ and $\phi_{q}$ are defined as the bond orientation and relative orientation, and *r_α_*, $\phi_{\hat{r}}^{\alpha }$ and $\phi_{q}^{\alpha }$ are the corresponding mean values to the *α*-peak, respectively. *σ_α_*, ${\eta_{\hat{r}}}^{\alpha }$ and $\eta_{q}^{\alpha }$ are the corresponding standard deviation and concentration parameters, and *I*_0_ is the modified Bessel function of the second kind and order 0 [26]. 

To simplify OPs, two kinds of the local OPs could be defined [47,48]. One is the OPs involving a certain molecule *i*, and the other is the averaged value within a divided simulation cell (*C*), given by Equations (3) and (4), respectively [26]:

(3) $$\theta_{i}^{*}=\sum_{\alpha }\varphi_{i,\alpha }^{*}$$

(4) $$\theta_{C}^{*}=\frac{1}{N_{C}}\sum_{i\in C}\sum_{\alpha }\varphi_{i,\alpha }^{*}$$

where “*” represents “db” and “dr” in Equations (1) and (2). 

### 2.2. Finite Temperature String

According to Maragliano et al. [33], string is a technique used to determine the minimum energy path or MFEP by locally evaluating the mean force and tensor. 

Given a set of *N* collective variables, *θ*(*x*) = (*θ*_1_(*x*), *θ*_2_(*x*), …, *θ_N_*(*x*)), where *x* ∈ *R^n^* are the Cartesian coordinates, the function of the free energy depending on *z* = (*z*_1_, *z*_2_, …, *z_N_*) could be defined as $F\left(z\right)=-k_{B}T\ln\left(Z^{-1}\int_{R^{n}}e^{-\beta V\left(x\right)}\delta \left(z_{1}-\theta_{1}\left(x\right)\right)\ldots \delta \left(z_{N}-\theta_{N}\left(x\right)\right)dx\right)$, in which *V*(*x*) is the potential energy, $Z=\int_{R^{n}}e^{-\beta V\left(x\right)}dx$ and *β* = 1/*k_B_T*, *k*_B_ and *T* are the Boltzmann constant and temperature, respectively. 

For a string denoted by *z*(α, *t*) at the evolutionary time *t*, where α ∈ [0, 1] is used to parametrize the string, the evolution equation involving the free energy gradient to make it converge to MFEP could be given by

(5) $$\frac{\partial z_{i}\left(\alpha ,t\right)}{\partial t}=-\sum_{j,k=1}^{N}P_{ij}\left(\alpha ,t\right)M_{jk}\left(z\left(\alpha ,t\right)\right)\frac{\partial F\left(z\left(\alpha ,t\right)\right)}{\partial z_{k}}$$

where *P_ij_*(α, *t*) is the projector on the plane perpendicular to the path at *z*(α, *t*), *M_jk_*(*z*(α, *t*)) is the tensor, and $\frac{\partial F\left(z\left(\alpha ,t\right)\right)}{\partial z_{k}}$ (i.e., ∇F(z)) is the free energy gradient (i.e., mean force). Given two minima of the free energy located at *z_a_* and *z_b_*, Equation (5) can be solved according to the boundary conditions *z*(0, *t*) = *z_a_* and *z*(1, *t*) = *z_b_*. As *t→*∞ the solution of (5) converges to a MFEP connecting *z_a_* and *z_b_*, and simultaneously the tangent vector is parallel to M(z)∇F(z) at every point z, i.e.,

(6) $$0=\sum_{j,k=1}^{N}P_{ij}\left(\alpha \right)M_{jk}\left(z\left(\alpha \right)\right)\frac{\partial F\left(z\left(\alpha \right)\right)}{\partial z_{k}}$$

where, $P_{ij}\left(\alpha \right)=\delta_{ij}-{\hat{t}}_{i}\left(\alpha \right){\hat{t}}_{j}\left(\alpha \right)$, ${\hat{t}}_{i}\left(\alpha \right)=\frac{\partial z_{i}/\partial \alpha }{|\partial z_{i}/\partial \alpha |}\;$, and *M_jk_*(*z*) is defined as

(7) $$\begin{array}{l} M_{ij}\left(z\right)=Z^{-1}e^{\beta F\left(z\right)}\int_{R^{n}}\sum_{k=1}^{n}\frac{\partial \theta_{i}\left(x\right)}{\partial x_{k}}\frac{\partial \theta_{j}\left(x\right)}{\partial x_{k}}e^{-\beta V\left(x\right)}\delta \left(z_{1}-\theta_{1}\left(x\right)\right)\ldots \delta \left(z_{N}-\theta_{N}\left(x\right)\right)dx \end{array}$$

In other words, according to Maragliano et al. [33], as *t→*∞ the solution of (5) converges to a MFEP connecting *z_a_* and *z_b_* since Equation (6) is a stable fixed point of Equation (5). When any new points $z^{m}(t+\Delta t)$ satisfy $\left|z^{m+1}(t)-z^{m}(t)\right|=\left|z^{m^{\prime }+1}(t)-z^{m^{\prime }}(t)\right|$ to order $o(\Delta \alpha^{2})$ at time *t*, the string converges to the MFEP. Here, Δ*α* = 1/(*R* − 1), and *R* is the number of the discretization points in the string. 

Based on their estimators under the ergodicity with the large enough values of *k* and *T*, Equations (6) and (7) could be solved to obtain [33]:

(8) $$\frac{\partial F\left(z\right)}{\partial z_{j}}\approx \frac{k}{T}\int_{0}^{T}\left(z_{j}-\theta_{j}\left(x\left(t\right)\right)\right)dt$$

(9) $$M_{\mathrm{ij}}\left(z\right)\approx \frac{1}{T}\sum_{k=1}^{n}\int_{0}^{T}\frac{\partial \theta_{i}\left(x\left(t\right)\right)}{\partial x_{k}}\frac{\partial \theta_{j}\left(x\left(t\right)\right)}{\partial x_{k}}dt$$

Thus, a string could be evolved by the iteration of the mean force until convergence. 

### 2.3. Minimum Free-Energy Path from Finite Temperature String

(1) Initial Trajectory 

The first step is to generate an initial trajectory connecting two basins and obtain the corresponding initial OPs $\theta_{i,m}^{*}\left(0\right)$ and $\theta_{C,m}^{*}\left(0\right)$ using a MD simulation [55]. 

(2) K-means clustering to obtain a smooth initial string 

In order to obtain a smooth initial string, a K-means clustering algorithm was adopted. It consists of three steps: 

Step 1: Restricting sampling. In each independent space around each given representative point, the samples were taken by the harmonic functions:

(10) $$\psi \left(\theta_{C}^{db}\right)=\frac{k_{d}}{2}{\left[\theta_{C,m}^{d}-\theta_{C,m}^{d}\left(0\right)\right]}^{2}+\frac{k_{b}}{2}{\left[\theta_{C,m}^{b}-\theta_{C,m}^{b}\left(0\right)\right]}^{2}$$

(11) $$\psi \left(\theta_{C}^{dr}\right)=\frac{k_{d}}{2}{\left[\theta_{C,m}^{d}-\theta_{C,m}^{d}\left(0\right)\right]}^{2}+\frac{k_{r}}{2}{\left[\theta_{C,m}^{r}-\theta_{C,m}^{r}\left(0\right)\right]}^{2}$$

where *k^d^*, *k^b^* and *k^r^* are the spring coefficients, with the values of 5000.0 kcal/mol, 100.0 kcal/mol and 100.0 kcal/mol, respectively. 

Step 2: K-means clustering. 

In K-means clustering, the weight *ω*_*i*_ is given by:

(12) $$\omega_{i}=\rho_{i}\times s_{i}\times \frac{1}{a_{i}}$$

where *ρ_i_*, *s*_*i*_ and *a*_*i*_ mean the density of sample point *i* (i.e., the number of points *i* within a certain range), distance between different clusters or data set, and the distance between different sample points in the same cluster. They can be calculated by Equations (13)~(15) as follows:

(13) $$\rho_{i}=\sum_{j=1}^{n}f\left[d_{\omega }\left(x_{i},x_{j}\right)-\frac{1}{C_{n}^{2}}\sum_{i=1}^{n}\sum_{j=i+1}^{n}d_{\omega }\left(x_{i},x_{j}\right)\right]\;$$

(14) $$s_{i}=\{\begin{array}{c} \min\left(d_{\omega }\left(x_{i},x_{j}\right)\right),\rho_{j}<\rho_{i}\\ \max\left(d_{\omega }\left(x_{i},x_{j}\right)\right),\rho_{j}\leq \rho_{i} \end{array}\;$$

(15) $$a_{i}=\frac{2}{\rho_{i}\left(\rho_{i}-1\right)}\sum_{i=1}^{\rho_{i}}\sum_{j=i+1}^{\rho_{i}}d_{\omega }\left(x_{i},x_{j}\right)$$

where $d_{\omega }\left(x_{i},x_{j}\right)$ is the Euclidean distance, and $f\left(x\right)=\{\begin{array}{c} 1,x<0\\ 0,x\geq 0 \end{array}\;.$ The adjacent clustering centers with the maximum weights can be connected to form a temporary string. 

Step 3: Searching for a successive string via smooth function and obtaining a smooth initial string. 

Based on the successive string by the smooth function, OPs of the *m*th replica were calculated to evolve string, and thus a smooth initial string could be obtained.

(3) Determining the MFEP using SMCV method

The MFEPs can be determined with the SMCV method, as detailed in our previous investigations [47,48]. It consists of five steps: 

Step 1: Estimating the free energy gradient $\nabla_{z}F(z_{C,m}^{*})$ and metric tensor $M(z_{C,m}^{*})$ according to Equations (8) and (9), respectively.

Step 2: Calculating a new target OPs $z_{C,m(\mathrm{new})}^{*}$.

Step 3: Reparameterizing. Interpolate a curve *z*(α) through $z_{C,m(\mathrm{new})}^{*}$ via the b-spline fitting method, and compute the new targets OPs (signed as $z_{C,m+1}^{*}$) by interpolating to make the arc length at a constant between the consecutive replicas. 

Step 4: Computing the difference between $z_{C,m+1}^{*}$ and $z_{C,m}^{*}$, and judging convergence. 

Step 5: Based on the final converged string, calculating local OPs and PMF. 

**Scheme 1:**
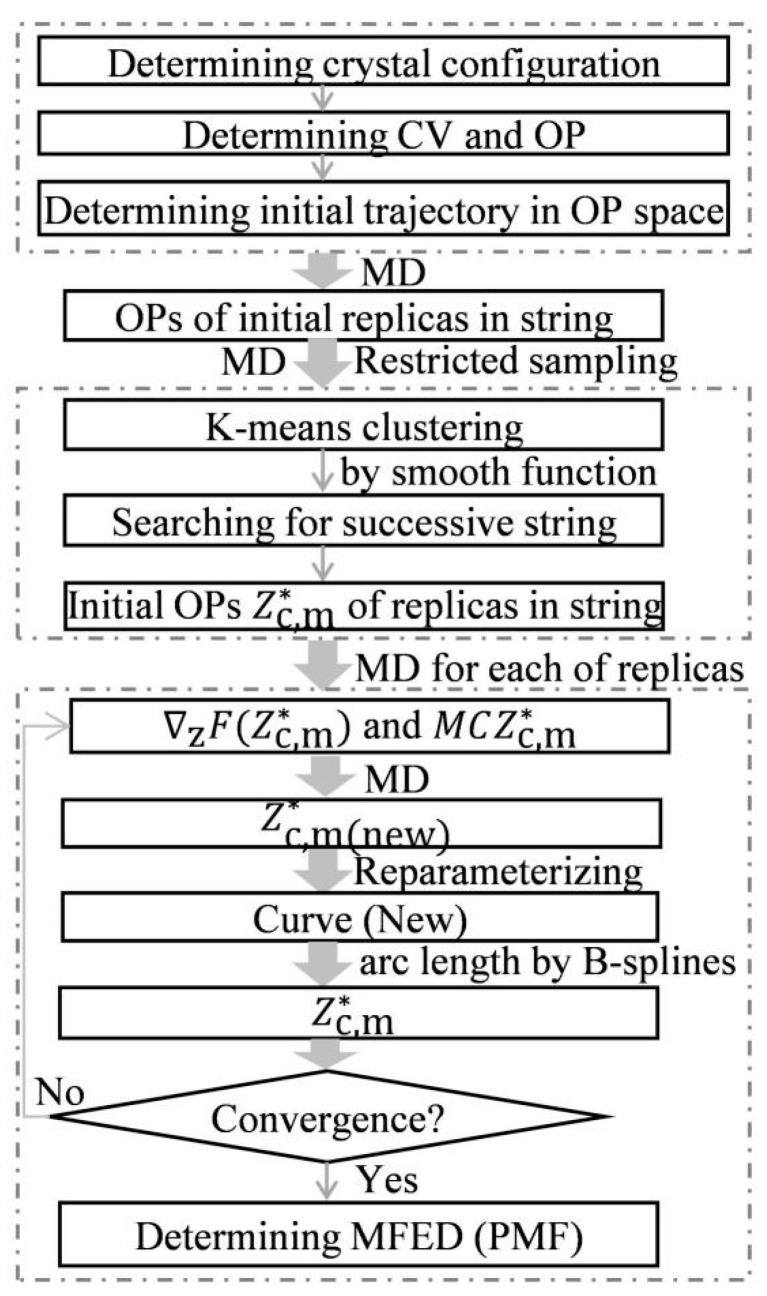
MD simulation process.

The MD simulation process can be shown in Scheme 1. 

### 2.4. External Electric Field Effect on Nucleation

The chemical potential difference between two crystal forms could be altered by the application of an external electric field, and thus the nucleation work could be modified, leading to an electric field effect on nucleation. 

The chemical potential difference Δ*μ*_ε_ under the electric field *E* is defined [56]:

(16) $$\Delta \mu_{\varepsilon }=\Delta \mu +\Delta c_{\varepsilon }E^{2}$$

where $\Delta c_{\varepsilon }=c_{\varepsilon (2)}-c_{\varepsilon (1)}$ is the difference in the permittivity between two crystal forms, and $c_{\varepsilon (2)}$ and $c_{\varepsilon (1)}$ are the relative permittivity of the product and initial crystal form, respectively. According to the classical nucleation theory and the literature [56], the nucleation work W* under the external electric field is as follows:

(17) $$W*=\frac{16A}{3\left(\Delta \mu +\Delta c_{\varepsilon }E^{2}\right)}$$

where *A* is a constant for a specific phase transition system. When $\Delta c_{\varepsilon }$ > 0, the chemical potential difference is enlarged and nucleation can be enhanced. In a situation where $\Delta c_{\varepsilon }$ < 0, the nucleus creation is impeded by the applied field, and nucleation is inhibited. If $\Delta c_{\varepsilon }$ = 0, the electric field does not influence nucleation. Therefore, according to the classical nucleation theory and the literature [56], the equation of the rate (*J*) for the nucleation under the external electric field can be written as

(18) $$J=C_{0}f^{*}\exp\left[-\frac{B}{\Delta \mu +c_{\varepsilon }E^{2}}\right]$$

where *C*_0_ and *B* are the constants for a specific phase transition system, and f* is the collision frequency of the particles on the surface of crystal nuclei (i.e., solid–liquid interface). 

## 3. Polymorphic Transformation of TNT

Due to *m*-TNT being more stable than *o*-TNT [54], only the polymorphic transformation from *o*-TNT to *m*-TNT was investigated without electric field. In order to verify the control of the electric field to the polymorphic transformation, the transformation from *m*-TNT to *o*-TNT was also present under the external electric field. 

### 3.1. Convergence of FTS and Polymorphic Transformation without Electric Field

(1) Peaks in pair distribution function 

**Figure 1:**
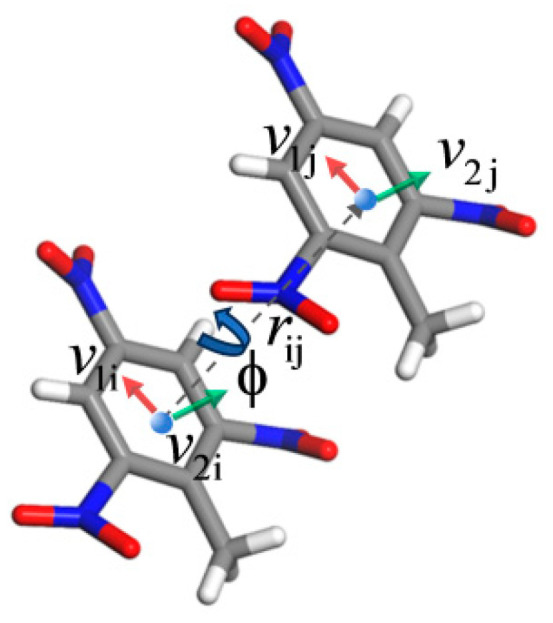
Construction of OPs based on pair distribution functions. Red, blue, gray and white represent O, N, C and H atoms, respectively.

Since the space group of *m*-TNT and *o*-TNT are *P2_1_/a* and *Pca2_1_*, characterized by two distinct molecular packing configurations with two conformationally unique molecules in the asymmetric unit with four asymmetric units in a unit cell [54] (see Figure S1), only three different peaks of the intermolecular distances could be found with a cutoff of 7.8 Å in the unit cell. Similar to Santiso et al. [26], the peaks in the pair distribution functions of *m*-TNT and *o*-TNT at 355 K were calculated (experimental temperature of 351 K [54]). These distributions were obtained by considering 2.5 ns MD simulations from the averaged values within the divided cells with the 3 × 3 × 3 grid for each of the systems with 216 molecules. The average peak locations and concentration parameters are shown in Table 1. For most of the structural parameters, the standard deviation is small and the concentration parameter is large. This displays the localization characteristics of the average centroid distance, bond orientation angle, and relative orientation angle distribution of the two crystal forms. Therefore, the bond distance OPs, bond orientation OPs and relative orientation OPs can be used to describe the polymorphic transformation of the TNT molecular crystal, and they are constructed (see Figure 1).

Compared with the reference structure, both the peak values of the pair distribution functions for *m*-TNT and *o*-TNT do not change significantly and for the two crystal forms, their peak values are very close. The high similarity between the structures of the two crystal forms makes it extremely difficult to construct a set of variables that can accurately describe their crystal transformation. Thus, during the evolution of strings, the free energy is difficult to converge. It will be discussed in detail below. 

(2) Convergence of FTS and K-means clustering 

**Figure 2:**
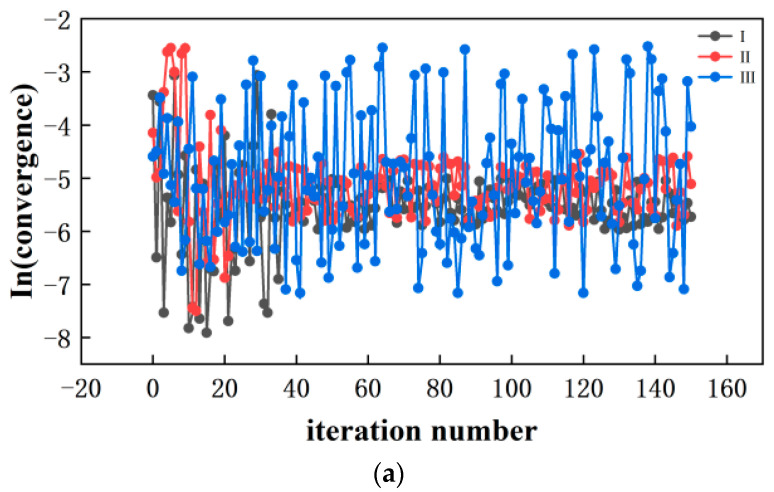

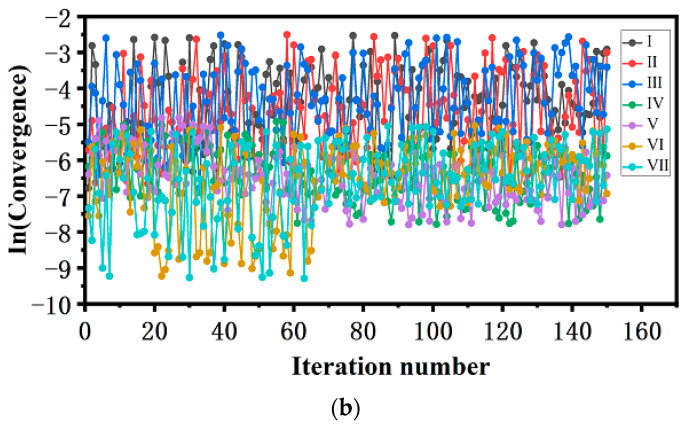
Convergence of the collective variables during the string evolution. (**a**) Convergence of collective variables for transformation from *o*-TNT to *m*-TNT without external electric field (I, II, and III represent the convergence of the bond orientation and relative orientation OPs obtained through K-means clustering sampling, and the relative orientation OPs obtained based on the average sampling, respectively). (**b**) Convergence of collective variables under the external electric field with the strengths of 5.14 × 10^8^ V/m (I, II and III represent the convergence of the bond orientation OPs obtained through K-means clustering sampling for the transformation from *o*-TNT to *m*-TNT along the fields parallel to the a-, *b*- and c-axes, respectively, and IV, V and VI represent those for the relative orientation OPs; VII is the convergence for the relative orientation OPs obtained through K-means clustering sampling for the transformation from *m*-TNT to *o*-TNT along the field parallel to the c-axis).

For the trajectories from the simulation times of 3 × 10 ns in 21 independent spaces, the average sampling and K-means clustering of OPs for bond orientation and relative orientation were performed. According to the definition of the convergence of the string shown by the literature [57], the values of convergence were calculated, and for clarity, the logarithmic values of convergence were determined with ln(convergence), as shown in Figure 2. I, II, and III represent the convergence of the bond orientation and relative orientation OPs obtained through K-means clustering sampling, and the relative orientation OPs obtained from the average sampling, respectively. It was found that for the bond orientation OPs, the ln(Convergence) values are large (−4.0~0.0). Those close to 0.0 may be caused by the “dimension explosion” of the OPs. For the relative orientation OPs with the average sampling, there is no convergence even after 150 iterations. The average sampling has significant dispersion and uncertainty. In this case, the simulation from a sampling space may cover adjacent spaces along the transition path, leading to an uneven initial string connecting adjacent points. As is well known, for the non-smooth string, there are always some independent sampling spaces that are not perpendicular to the current string, which causes the restricted samples around each representative point to interfere with each other and interweave together, leading to confusion in the evolution direction of the string. A smooth string means that these intermediate states can continuously change from one to the other, and the purpose of smoothing the string is to eliminate irrelevant information. It is found from I and II in Figure 2 that after the K-means clustering enhanced sampling, the strings of two OPs converge after 14 and 11 iterations, respectively. 

(3) Minimum free energy path 

PMF is the line integral of the restraint force close to the MFEP from the maximum likelihood in the space of collective variables [58]. 

**Figure 3:**
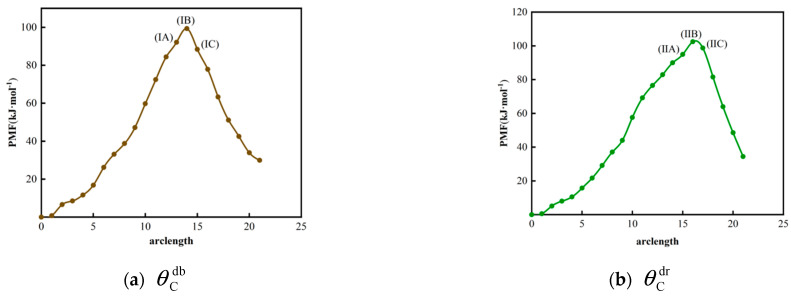

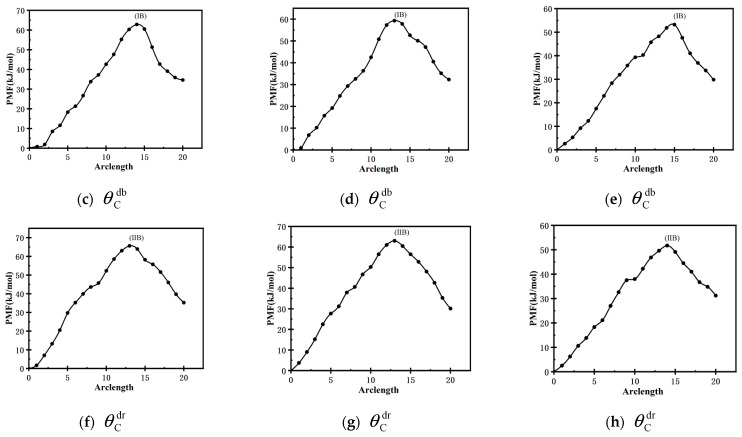
Curve of PMF with arc length ((**a**,**b**): PMF with $\theta_{C}^{\mathrm{db}}$ and $\theta_{C}^{\mathrm{dr}}$ for the polymorphic transformation without electric field, respectively; (**c**–**e**): PMF with $\theta_{C}^{\mathrm{db}}$ for the polymorphic transformation from *o*-TNT to *m*-TNT under the external electric fields with the strength of 51.40 × 10^8^ V/m along the *a*-, *b*- and *c*-axis, respectively; (**f**–**h**): PMF with $\theta_{C}^{\mathrm{dr}}$ for the polymorphic transformation from *o*-TNT to *m*-TNT under the external electric fields with the strength of 51.40 × 10^8^ V/m along the *a*-, *b*- and *c*-axis, respectively).

The convergent FTS path was obtained using the SMCV method. Figure 3a,b show the PMF curves as the function of the arclength along the converged FTS paths obtained from the SMCV method corresponding to the $\theta_{C}^{db}$ and $\theta_{C}^{dr}$ as the collective variables via K-means clustering, respectively. The difference in PMF between *o*-TNT and transition state is 102.5 and 99.5 kJ∙mol^−1^, and that of PMF between *o*-TNT and *m*-TNT is 34.3 and 30.1 kJ∙mol^−1^, respectively. Due to the PMF corresponding to the OP space of 27 dimensions (3 × 3 × 3) instead of one-dimensional coordinates, the values of PMF are higher than the real free energy barrier. 

**Figure 4:**
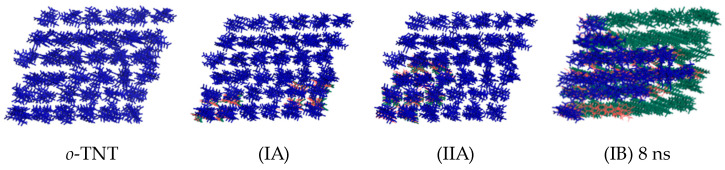

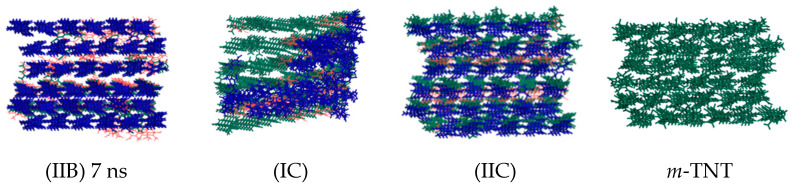
Changes in the local order parameters on the FTS path and the times for crystallization without electric field.

In order to gain further mechanistic insights into the polymorphic transformation, the local OPs of TNT were shown for the key snapshots at the points of the PFM curve along the trajectory going from *o*-TNT to *m*-TNT (see Figure 4). The molecules that can be regarded as *o*-TNT were labeled in blue with the values of $\theta_{C}^{db}$ > ~200 or $\theta_{C}^{dr}$ > ~220, and the green molecules were regarded as *m*-TNT with the values of $\theta_{C}^{db}$ < ~130 or $\theta_{C}^{dr}$ < ~150, whereas the wireframe molecules in red formed in the interface between the two polymorphs with the values in the range of 130~200 or 150~220 for $\theta_{C}^{db}$ and $\theta_{C}^{dr}$, respectively. (IB) and (IIB) represent the phase transition state, as the labels in Figure 3. 

From Figure 4, it can be seen that along the *o*-TNT → (IA) → (IIA) → (IB) → (IIB) → (IC) → (IIC) → *m*-TNT, the type of molecule gradually changes from the “all blue” initial conformation, the formation of the molecular clusters of which “Scattered green areas are surrounded by blue areas” (interface induction), to gradually increasing “sporadic green” molecular clusters (i.e., local initiation, multinuclear asynchronous generation and increase), and then to the “blue being engulfed by green” conformation. Finally, a “complete green color” is formed. This indicates that the polymorphic transformation between *o*-TNT and *m*-TNT is an interface induced, locally initiated, and multi-nucleus asynchronous development process, which is consistent with the results of the polymorphic transformation of the molecular crystal from Trout et al. [23].

**Figure 5:**
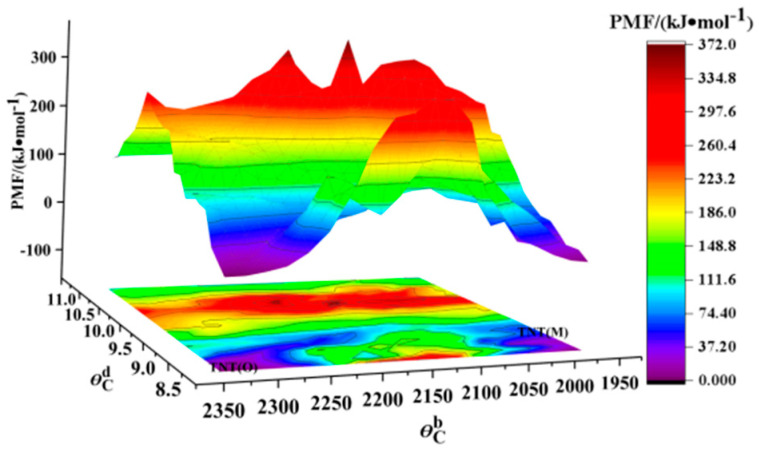
Three-dimensional diagram of PMF along FTS path with global order parameters.

The PMF along the FTS path with the global OPs is shown in Figure 5. The value of $\theta_{C}^{d}$ is approximately 10.5. The polymorphic transformation between *o*-TNT and *m*-TNT is mainly controlled by $\theta_{C}^{b}$. 

### 3.2. Polymorphic Transformation of TNT under the External Electric Fields

(1) Peaks in pair distribution function under the external electric fields 

For the polymorphic transformation of the molecular crystals, the strengths of the external electric fields were often in the range of 1.0 × 10^8^ V/m~102.80 × 10^8^ V/m [56]. Therefore, in this work, the external electric field with the strength of 51.40 × 10^8^ V/m was used to investigate the polymorphic transformation of TNT. The average peak locations and concentration parameters under the external electric fields along the directions of the *a*-, *b*- and *c*-axes are shown in Table 2. Compared with the values without the external electric fields (see Table 1), both the peak values and the concentration parameters of the pair distribution functions for *o*-TNT and *m*-TNT do not change obviously. Moreover, there is no significant pattern where the effect of external electric fields in any direction on the structure is more significant than that in the other directions. This is inconsistent with our recent investigation on the effect of external electric fields on the structures of the ionic crystal TKX-50 [48]. It is noted that, compared to the ionic crystals, the dipole moment of the molecules in molecular crystals is much smaller, which results in a much smaller response to the external electric fields, with the small effect on the structure in the molecular crystal. 

(2) Polymorphic transformation from *o*-TNT to *m*-TNT under external electric field

From Figure 2b, similar to those without the external electric field for the trajectories from the simulation of 3 × 10 ns in 21 independent spaces under the external electric fields with the strength of 51.40 × 10^8^ V/m, there is no convergence even after 150 iterations for the average sampling of OPs for the bond orientation or relative orientation, while the convergence of the bond orientation and relative orientation OPs through K-means clustering sampling was obtained after 80 iterations. As mentioned above, their convergences were found after 14 and 11 iterations without an electric field. This result shows that the external electric field does not promote the convergence of sampling for Ops; on the contrary, it even makes the convergence worse. Only by performing K-means clustering can the convergence be obtained with a significant improvement. 

When the $\theta_{C}^{db}$ as the collective variables, the difference in PMF between the *o*-TNT and transition state is 62.8, 59.3 and 53.2 kJ∙mol^−1^ under the external electric fields with the strength of 51.40 × 10^8^ V/m along the *a*-, *b*- and *c*-axes, respectively (See Figure 3c–h). As for the $\theta_{C}^{dr}$, they are 65.6, 63.0 and 51.7 kJ∙mol^−1^, respectively. The difference in PMF between the *o*-TNT and transition state is decreased in comparison with those in no field. Moreover, the fields parallel to the *c*-axis affect the difference in PMF between the *o*-TNT and transition state of TNT more than those parallel to the *a*- or *b*-axes. It is well known that, for the molecular crystal, the polymorphic transformation process mainly includes the rearrangement of both intra- and inter-molecular degrees of freedom, which is closely related to the intermolecular interaction. From Figure S1f, the molecules are in the π∙∙∙π stacked in layers along the *c*-axis, while along the *a*- and *b*-axis, the H-bonding interactions are formed. The H-bonding interactions are strengthened by the increased dipole moments more significantly than the intermolecular π∙∙∙π stackings along the *c*-axes under the external electric fields, since the π∙∙∙π stackings themselves are weaker than the H-bonding interactions. The weaker the intermolecular interaction, the more notable the change is under the external electric fields, leading to the more active transition state and thus the smaller difference in PMF between *o*-TNT and transition state of TNT. This may be why the fields parallel to the *c*-axis affect the difference in PMF between the *o*-TNT and transition state of TNT more than those parallel to the *a*- or *b*-axes. Therefore, applying an external electric field along the c-axis direction has more practical value for achieving the polymorphic transformation from *o*-TNT to *m*-TNT. 

**Figure 6:**
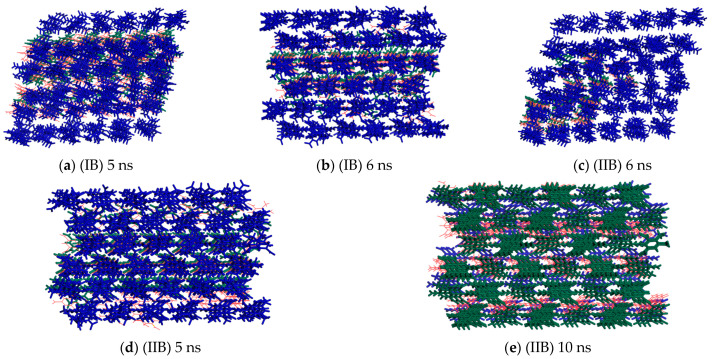
Changes in local order parameters on the FTS path and the times for crystallization under the external electric fields with the strength of 51.40 × 10^8^ V/m. ((**a**,**c**,**e**): electric fields parallel to the *c*-axis, (**b**,**d**): electric fields parallel to the *b*-axis. (**a**–**d**): polymorphic transformation from *o*-TNT to *m*-TNT; (**e**): polymorphic transformation from *m*-TNT to *o*-TNT).

For the polymorphic transformation under the external electric fields with the strength of 51.40 × 10^8^ V/m, the local OPs of TNT were also shown for the key snapshots at the points of the PFM curve along the trajectory going from *o*-TNT to *m*-TNT (see Figure 6). Similar to those without the electric fields, along the *o*-TNT → (IB) or (IIB) → *m*-TNT, the type of molecule gradually changes from the “all blue” initial conformation to the “complete green color”, indicating a complete polymorphic transformation from the *o*-TNT to *m*-TNT crystal form under the external electric field. This indicates that, under the external electric field, the polymorphic transformation between *o*-TNT and *m*-TNT is also an interface induced, locally initiated, and multi-nucleus asynchronous process. 

However, from Figure 4 and Figure 6, an interesting phenomenon was found: the time of the polymorphic transformation (i.e., the time from *o*-TNT to IB or IIB) is reduced when the external electric field is applied in comparison to its absence. The times of the polymorphic transformation shown by the bond orientation and relative orientation OPs are 8 ns and 7 ns without the electric field, while under the external electric field along the lattice *c*-, and *b*-axes with the strengths of 51.40 × 10^8^ V/m, the corresponding times are reduced by 5 ns and 6 ns, respectively. From the experiments, Radacsi [59] has observed that the crystals grew in less time under the application of the electric field in comparison to those grown without the electric field. The crystal growth rate increased from 8.3 μm/min to 126 μm/min. From the recrystallization of isonicotinamide, Hammadi et al. [60] proposed that the control of crystallization shown by the induction time was attributed to the increased local supersaturation due to the electromigration by the application of the electric field. 

(3) Polymorphic transformation from *m*-TNT to *o*-TNT under external electric field

Different from the polymorphic transformation from *o*-TNT to *m*-TNT, for the transformation from *m*-TNT to *o*-TNT, there is no convergence after 200 iterations for the sampling of the bond orientation OPs through K-means clustering under the external electric fields with the strength of 51.40 × 10^8^ V/m. For the relative orientation OPs through K-means clustering sampling, the convergence was obtained after 60 iterations (see Figure 2b). 

Although the convergence and formation of molecular clusters of which “Scattered green areas are surrounded by blue areas” along the polymorphic transformation from *m*-TNT to *o*-TNT were found (see Figure 6e), the complete path was not discovered with our significant efforts. This indicates that, under the external electric field, the polymorphic transformation path from *m*-TNT to *o*-TNT is not working. This phenomenon has also been confirmed experimentally for the polymorphic transformation of the molecular crystals. For example, when no electric field was applied to the solution, the isonicotinamide crystals grown were the metastable form I [59]. However, when a homogeneous electric field of 5.6 × 10^5^ V/m was applied, the crystals formed on the anode were of the stable form II. Moreover, the author observed that when an inhomogeneous electric field was lower than 5.6 × 10^5^ V/m, a combination of both polymorphs forms was present. The study found that raising the strength of the field led to the transformation of the polymorphic structure from the metastable form I to the stable form II, and thus had the ability to alter the crystallization kinetics. Mirkin et al. [61] also detected that there appeared to be a favored polymorphic transformation orientation under the electric field for the lysozyme crystal, as was confirmed using X-ray crystallography that the structure of the thaumatin crystals was not altered by the application of the internal electric field. Therefore, under the external electric field, only the polymorphic transformation from *o*-TNT to *m*-TNT will occur, while the transformation from *m*-TNT to *o*-TNT will be prevented. The polymorphic transformation orientation of the molecular crystals could be controlled using the external electric field, and an applied electric field has the potential to be used for controlling polymorphic formation in crystallization processes.

(4) Explanation of transformation orientation controlled using external electric field

For the favored polymorphic transformation orientation of molecular crystals controlled using the external electric field, Aber et al. [62] claimed that this was a consequence of the orientation of pre-established groups of molecules brought about by the electric field, aiding their formation into a crystalline arrangement. Profio et al. [63] proposed that the rates of nucleation had a leading part in shaping the polymorphic outcome, and the molecular nucleation process in the rate-limiting stage acted as the foundations for the certain polymorphic form. To further reveal the essence of the transformation orientation controlled using the external electric field, i.e., the phenomenon that the polymorphic transformation from *o*-TNT to *m*-TNT occurs while that from *m*-TNT to *o*-TNT is prevented, the dipole moment of TNT was calculated (see “Supporting Information”), and the corresponding permittivity, nucleation work and rate for the nucleation were discussed under the external electric field with the strength of 51.40 × 10^8^ V/m along the *c*-axis. 

The total dipole moments of a single molecule in the fixed direction in the *o*-TNT and *m*-TNT polymorphism are 1.4320 and 1.4568 Debye under the external electric field with the strength of 51.40 × 10^8^ V/m along the c-axis direction at the M06-2X/aug-cc-pVTZ level, respectively. According to the QSPR (Quantitative Structure–Property Relationships) model of the permittivity and dipole moment for the organic compounds based on the neural network methods by Schweitzer [64], the larger the dipole moment, the greater the permittivity is. This indicates that the permittivity *ε* of the molecule in *m*-TNT is larger than that in the *o*-TNT under the external electric field. Thus, for the polymorphic transformation from *o*-TNT to *m*-TNT, the relative permittivity $\Delta c_{\varepsilon }$ from the product and initial crystal forms (i.e., $\Delta c_{\varepsilon }=c_{\varepsilon (m-\mathrm{TNT})}-c_{\varepsilon (o-\mathrm{TNT})}$) was enhanced ($\Delta c_{\varepsilon }$ > 0). According to Equations (16)~(18), the chemical potential difference was enlarged, and the nucleation work W* was reduced and the rate (*J*) for the nucleation was enhanced. Therefore, the polymorphic transformation from *o*-TNT to *m*-TNT was promoted under the external electric field. On the contrary, for the polymorphic transformation from *m*-TNT to *o*-TNT, $\Delta c_{\varepsilon }$ < 0, and the chemical potential difference was reduced, and the nucleation work W* was enlarged and the rate for the nucleation was reduced, leading to an inhibited nucleation under an external electric field. 

(5) Relationship between PMFs and field strengths

**Figure 7:**
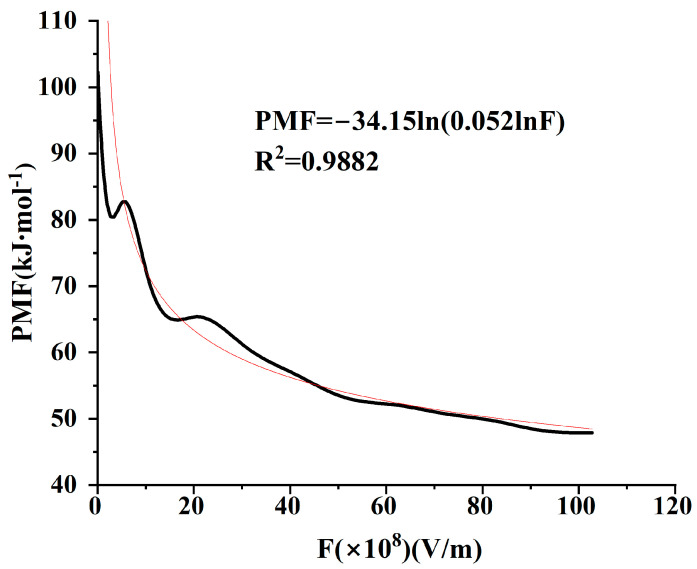
Field strengths vs. changes in PMF between *o*-TNT and transition state.

In order to further verify the influence of the external electric field along the c-axis direction on the polymorphic transformation from *o*-TNT to *m*-TNT, the relationship between the changes in the difference in PMF between *o*-TNT and transition state and field strengths has been studied. The field strengths are 0.00, 0.514 × 10^8^, 5.14 × 10^8^, and 10.28 × 10^8^~102.80 × 10^8^ V/m with a step of 10.28 × 10^8^ V/m. From Figure 7, a linear result (*R*^2^ = 0.9882) was shown by Equation (19):

PMF = −34.15ln(0.052ln*F*) (19)

The *R*_2_ value is relatively low. Noting this, under the external electric field, the PMF value is not only related to the phase transition of *o*-TNT, but also to the change in transition state. Their changes are complex and possible asynchronous, which leads to a relative low *R*_2_ value. This linear result indicates that the external electric fields (*F*) have an influence on the polymorphic transformation from *o*-TNT to *m*-TNT. This suggests that the process of the polymorphic transformation can be controlled by changing the external electric fields. 

## 4. MD Simulation Details

According to the experimental data, the *o*-TNT (CCDC: 227800, *a* = 14.910(2) Å, *b* = 6.034(2) Å, *c* = 19.680(4) Å, *β* = 90.0°, Pca2_1_) and *m*-TNT (CCDC: 227799, *a* = 14.911(1) Å, *b* = 6.034(1) Å, *c* = 20.882(3) Å, *β* = 110.365(1)°, P2_1_/a) crystal structures [54] were constructed with the size of 6 × 6 × 6 (216 molecules) (see Figure S1). Based on the molecular structure and strategy of the OP construction for molecular crystal [26], a set of OPs for TNT were built with the pair distribution function (see Figure 1). 

Due to the more stability of *m*-TNT compared to *o*-TNT, the polymorphic transformation from *o*-TNT to *m*-TNT was mainly discussed in this study. The polymorphic transformation mainly involves the disruption of the cohesive forces in the *o*-TNT crystal lattice and the formation of new cohesion forces in *m*-TNT. Due to the slight methyl rotation, the internal degrees of freedom of the conformational transformation can be ignored. Based on molecular symmetry and point molecule representation [55], two vectors *v*_1_ and *v*_2_ can be used to represent molecular orientation, where the former points from the center of the TNT hexagonal ring towards the carbon atom connected to the 4-nitro group, while the latter is perpendicular to the plane of the TNT hexagonal ring. The bond orientation $\phi_{\hat{r}}$ is defined as the projection of the *i*-th molecule *v*_2i_ onto the centroid vector ${\hat{r}}_{ij}$; the relative direction displays the rotation to, as shown in Figure 1. 

In order to obtain the OPs, firstly a 1.0 ns MD simulation was carried out on the system with the 216 *o*-TNT and *m*-TNT molecules at 355 K and 1 bar, respectively. The particle mesh Ewald method was used to model the periodic electrostatic interactions and a Langevin hot bath with an oscillation frequency of 25 ps^−1^ was used to control the temperature. After equilibrium, the annealing treatment and energy minimization relaxation were carried out at a constant volume and zero temperature. Then, a 2.5 ns MD simulation was carried out at 355 K with a time step of 0.5 fs by using a MS 8.0 software package. Based on the obtained trajectory and Equations (3) and (4) in the case of the simulated box with the 3 × 3 × 3 grid, the OPs were determined using the maximum likelihood estimation method. 

For the MD simulation of FTS evolution, the ensemble remains at 355 K and 1 bar. The first step for constructing FTS is to generate an initial trajectory connecting two basins. It is very difficult for the polymorphic transformation of the organic small molecular crystals due to the size effects of system and dimensionality of the OP space. After many repeated processes, a trajectory initiated from the *o*-TNT to *m*-TNT basin with 216 molecules was found to transform into ε-CL-20 during several ns, judged by the ratio of the b to c lattice vectors. Thus, an initial string consisting of the selected 21 replicas, including the *o*-TNT and *m*-TNT basins, was obtained according to the approximately equal b/c step size. The effectiveness of this method has been verified by our group [44]. Then, each of the initial replicas were optimized with the fixed b/c value, and the corresponding initial OPs were calculated using Equations (3) and (4), using a MD simulation at 355 K with the NVE ensemble. To accelerate convergence, we chose each initial configuration closest to the new target OP from the previous step. To maintain the stability of simulation, the center of the OP constraint is moved from that in the previous step to a new value in the previous period of time. The constraint force is averaged for each step in the later period. The initial velocity is randomly assigned from the Maxwell–Boltzmann distribution. The CHARMM22 force field was selected with the calculations using a NAMD software package [65]. 

The external electric fields were applied along the lattice *a*-, *b*-, and *c*-axis, respectively, with the strength of 0.514 × 10^8^, 5.14 × 10^8^, and from 10.28 × 10^8^ to 102.80 × 10^8^ V/m with a stepsize of 10.28 × 10^8^ V/m by using an LAMMPS software package. Based on the obtained trajectory, the OPs were determined using the maximum likelihood estimation method. The following calculations are the same as those with MS and NAMD software packages, and the results from FTS evolution are in Table 1 and Table 2, and Figure 1, Figure 2, Figure 3, Figure 4, Figure 5, Figure 6 and Figure 7 and Figure S1. The dipole moments of molecules under the external electric field with the strength of 51.40 × 10^8^ V/m at the M06-2X/aug-cc-pVTZ level by using Gaussian 09 programs [66].

## 5. Conclusions

In this work, two kinds of the local OPs of TNT were constructed, and the MFEP for the polymorphic transformations between *o*-TNT and *m*-TNT were sketched using SMCV method with the K-means clustering to sampling of OPs under the external electric fields. 

(1) The K-means clustering algorithm based on Euclidean distance and density weight can be used for the enhanced sampling of the OPs in the investigation of the polymorphic transformation for molecular crystal, which can improve the conventional FTS method and enable fast convergence of free energy. The FTS method based on the average-based sampling is unfavorable to the iteration and convergence of string for the polymorphic transformation between the molecular crystal *o*-TNT and *m*-TNT. 

(2) The polymorphic transformation could be regarded as a process of the nucleation of *o*-TNT within the *m*-TNT phase, and a surface-mediated nucleation mechanism was confirmed. 

(3) The difference in PMF between the *o*-TNT and transition state is decreased in comparison with those in no field. Moreover, the fields parallel to the *c*-axis affect the difference in PMF between *o*-TNT and transition state of TNT more than those parallel to the *a*- or *b*-axes. 

(4) The external electric field does not promote the convergence of sampling for OPs; on the contrary, it actually makes the convergence worse. Only by performing K-means clustering can the convergence obtain a significant improvement. Furthermore, the time of the polymorphic transformation is reduced when the external electric field is applied in comparison to its absence. Moreover, under the external electric field, only the polymorphic transformation from *o*-TNT to *m*-TNT occur, while the transformation from *m*-TNT to *o*-TNT is prevented. Due to the larger dipole moment of a single molecule in *m*-TNT than that in *o*-TNT under the external electric field, the permittivity *ε* of the molecule in *m*-TNT is larger than that in *o*-TNT. Thus, for the polymorphic transformation from *o*-TNT to *m*-TNT, the relative permittivity $\Delta c_{\varepsilon }$ was enhanced, the chemical potential difference was enlarged, and the nucleation work W* was reduced and the rate (*J*) for the nucleation was enhanced. Therefore, the polymorphic transformation from *o*-TNT to *m*-TNT was promoted under the external electric field. On the contrary, for the polymorphic transformation from *m*-TNT to *o*-TNT, $\Delta c_{\varepsilon }$ < 0, and the nucleation work W*, was enlarged and the rate for the nucleation was reduced, leading to an inhibited nucleation under external electric field. Therefore, the polymorphic transformation orientation of the molecular crystals could be controlled using the external electric field, and an applied electric field has the potential to be used for controlling polymorphic formation in crystallization processes. 

(5) The relationship between the changes in PMF and field strengths was found, suggesting that the process of the polymorphic transformation can be controlled by changing the external electric fields. 

This work provides an effective way to explore the polymorphic transformation of the molecular crystals at the molecular level, and it is useful to realize the control of the production process and the improvement of the properties of the energetic materials by using the external electric fields. 

## Figures and Tables

**Table 1:** Average peak locations and concentrations parameters for the *o*-TNT and *m*-TNT crystals at 355 K.

Sample	*r*(Å)	1/*σ*^2^(Å^−1^)	$\phi_{\hat{r}}^{\alpha }$(°)	$\eta_{\hat{r}}^{\alpha }$	$\phi_{q}$(°)	$\eta_{q}^{\alpha }$
	5.68(5.65) ^a^	22.03	74.67(71.53)	23.18	10.55(12.31)	8.93
*o*-TNT	6.22(6.26)	19.28	68.43(66.28)	11.53	57.79(56.29)	15.62
	6.68(6.67)	28.32	49.62(53.10)	19.18	85.62(84.17)	13.17
	5.63(5.64)	61.25	41.18(42.53)	22.36	172.88(169.17)	19.32
*m*-TNT	5.72(5.65)	9.02	85.37(82.11)	18.52	163.27(165.02)	15.78
	7.43(7.46)	11.25	51.28(53.62)	21.87	44.50(45.13)	27.62

^a^ The peak values of the pair distribution function of the reference structure calculated at 355 K temperature are shown in parentheses.

**Table 2:** Average peak locations and concentrations parameters for the *o*-TNT and *m*-TNT crystals at 355 K under the external electric field with the strength of 51.40 × 10^8^ V/m.

	*r*(Å)	1/*σ*^2^(Å^−1^)	$\phi_{\hat{r}}^{\alpha }$(°)	$\eta_{\hat{r}}^{\alpha }$	$\phi_{q}$(°)	$\eta_{q}^{\alpha }$
*o*-TNT	5.67 ^a^ (5.67) ^b^ *5.79* ^c^	24.18(25.33) *27.62*	67.82(65.31) *62.16*	20.23(20.56) *23.51*	8.12(6.62) *9.93*	7.88(8.39) *6.26*
	6.25(6.24) *6.26*	17.63(19.81) *15.17*	65.17(62.76) *69.35*	13.18(9.03) *17.63*	53.16(51.87) *49.27*	13.98(13.25) *16.07*
	6.67(6.63) *6.65*	26.55(24.89) *20.12*	52.88(57.13) *49.21*	22.56(24.12) *27.80*	82.35(86.18) *76.22*	15.26(17.53) *19.18*
*m*-TNT	5.66(5.63) *5.92*	71.37(75.28) *63.12*	46.26(49.17) *50.89*	20.15(23.62) *20.83*	141.40(123.79) *165.18*	21.52(24.31) *20.17*
	5.70(5.71) *5.73*	11.18(15.62) *13.79*	67.28(65.39) *61.04*	16.31(18.15) *16.27*	149.01(153.82) *157.25*	18.92(20.13) *17.65*
	7.46(7.42) *7.45*	14.31(13.29) *12.10*	55.97(51.18) *60.12*	19.31(21.83) *16.72*	45.23(46.18) *40.35*	25.31(27.02) *24.85*

^a^ The values of the average peak locations and concentrations parameters corresponding to the positive direction of the *a*-axis. ^b^ The values of the average peak locations and concentrations parameters (in parentheses) corresponding to the positive direction of the *b*-axis. ^c^ The values of the average peak locations and concentrations parameters (in italic) corresponding to the positive direction of the *c*-axis.

## Data Availability

The data related to this research can be accessed upon reasonable request via email.

## Supplementary Materials

The following supporting information can be downloaded at: https://www.mdpi.com/article/10.3390/molecules29112549/s1. Figure S1: Important lattice planes of o-TNT and m-TNT with the crystal size of 6 × 6 × 6 (216 molecules).
