# Supplementary material for: Finite Temperature String with Order Parameter as Collective Variables for Molecular Crystal: A Case of Polymorphic Transformation of TNT under External Electric Field
Source: Molecules. 2024 May 29;29(11):2549. doi: 10.3390/molecules29112549 (PMC11173574; doi:10.3390/molecules29112549)
Supplement: Supplementary file 1 [file molecules-29-02549-s001.zip › molecules-2999487-supplementary.pdf]

Supporting Information

Finite Temperature String with Order Parameter as Collective Variables for Molecular Crystal: A Case of Polymorphic Transformation of TNT under External Electric Field

Shi-jie Niu and Fu-de Ren

Figure S1. Important lattice planes of *o*-TNT and *m*-TNT with the crystal size of 6×6×6 (216 molecules).

Calculation method of dipole moment.

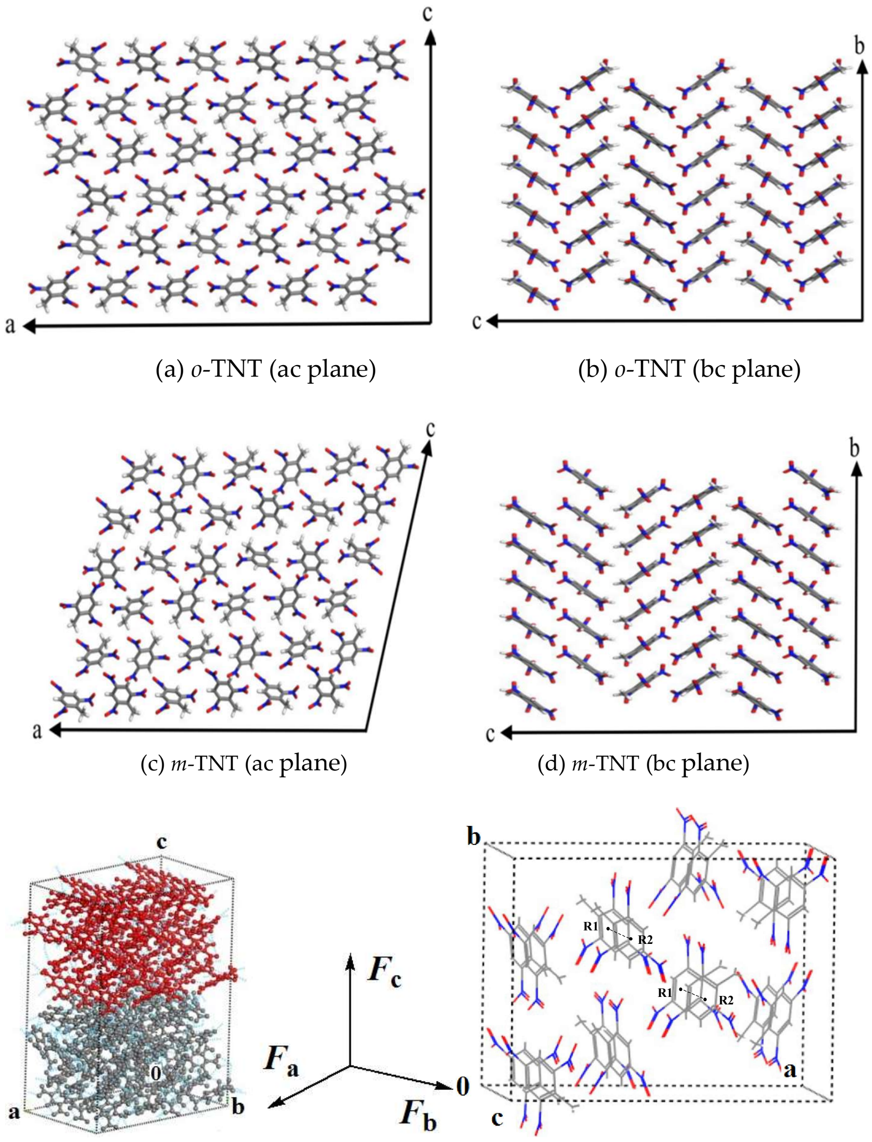

(a) *o*-TNT (ac plane) (b) *o*-TNT (bc plane)

(c) *m*-TNT (ac plane) (d) *m*-TNT (bc plane)

(e) Configuration under external electric field (f) π···π stacking along *c*-axis

Figure S1. Important lattice planes of *o*-TNT and *m*-TNT with the crystal size of 6×6×6 (216 molecules). In (e), two-phase configuration of TNT after the 500 ps NPT simulation at 470 K. F_a, F_b and F_c with the arrows mean the orientations of the external electric fields along the lattice *a*-, *b*-, and *c*-axes, respectively. In (f), the molecules are stacked in layers along the *c*-axis (R1···R2 represents the π···π stacking). The red and grey molecules indicate two-phase configuration.Red, blue, Gray and white represent O, N, C and H atoms, respectively.

Calculation method of dipole moment

To further reveal the essence of the transformation orientation controlled by external electric field, i.e., the phenomenon that the polymorphic transformation from *o*-TNT to *m*-TNT occurs while that from *m*-TNT to *o*-TNT is prevented, the dipole moment of TNT was calculated.

Firstly, one molecule was "dug out" from experimental crystals *o*-TNT and *m*-TNT in the literature [54], respectively. In order to simulate the effect of an external electric field along the c-axis of the crystal on the molecular dipole moment, two molecules were not optimized, and the orientation of the molecules remained consistent with their orientation in the crystal. The direction of the electric field also remained consistent with the c-axis direction of the crystal. Then, the dipole moments of them under the external electric field with the strength of 51.40×10^{8} V/m at the M06-2X/aug-cc-pVTZ level by using the Gaussian 09 programs [66].

[66] Frisch MJ, Trucks GW, Schlegel HB, Scuseria GE, Robb MA, Cheeseman JR, Scalmani G, Barone V, Mennucci B, Petersson GA, Nakatsuji H, Caricato M, Li X, Hratchian HP, Izmaylov AF, Bloino J, Zheng G, Sonnenberg JL, Hada M, Ehara M, Toyota K, Fukuda R, Hasegawa J, Ishida M, Nakajima T, Honda Y, Kitao O, Nakai H, Vreven T, Montgomery JA, Jr, Peralta JE, Ogliaro F, Bearpark M, Heyd JJ, Brothers E, Kudin KN, Staroverov VN, Kobayashi R, Normand J, Raghavachari K, Rendell A, Burant JC, Iyengar SS, Tomasi J, Cossi M, Rega N, Millam JM, Klene M, Knox JE, Cross JB, Bakken V, Adamo C, Jaramillo J, Gomperts R, Stratmann RE, Yazyev O, Austin AJ, Cammi R, Pomelli C, Ochterski JW, Martin RL, Morokuma K, Zakrzewski VG, Voth GA, Salvador P, Dannenberg JJ, Dapprich S, Daniels AD, Farkas O, Foresman JB, Ortiz JV, Cioslowski J, Fox DJ, Gaussian 09, Inc.. USA: Wallingford CT, 2009.
